# Molecular mechanism and intervention measures of microvascular complications in diabetes

**DOI:** 10.1515/med-2023-0894

**Published:** 2024-04-17

**Authors:** Rui Xu, Ziming Fang, Hongyu Wang, Ye Gu, Liying Yu, Boyang Zhang, Jingyu Xu

**Affiliations:** Hanan Branch of the Second Affiliated Hospital of Heilongjiang University of Chinese Medicine, Harbin, China; The First Affiliated Hospital of Heilongjiang University of Traditional Chinese Medicine, Harbin, China; Dongning Maternal and Child Care Service Center, Mudanjiang, China; Heilongjiang University Of Chinese Medicine, Harbin, China; Daqing Traditional Chinese Medicine Hospital, Daqing, China; Wuxi Traditional Chinese Medicine Hospital, Wuxi, China; Department of Cardiology, The First Affiliated Hospital of Heilongjiang University of Traditional Chinese Medicine, Harbin, China

**Keywords:** microvascular complication, epidemiology, molecular mechanism, risk factors, treatment

## Abstract

**Objective:**

In this article, the epidemiology, molecular mechanism of occurrence and development, risk factors, and treatment of diabetic microvascular complications such as diabetic nephropathy, diabetic retinopathy, and diabetic peripheral neuropathy were discussed, providing the theoretical basis for more accurate elucidation of the pathogenesis and treatment of diabetic microvascular complications.

**Methods:**

The electronic database of PubMed was searched, and retrieved papers were screened for eligibility by two independent reviewers. Data were extracted using a standardized data extraction form and the quality of included papers was assessed.

**Results:**

Thirty-eight articles were included. Diabetes nephropathy, diabetes peripheral neuropathy, and diabetes retinopathy are the most common and serious microvascular complications of diabetes in clinical patients. Renin–angiotensin system blockers, beta drugs, statins, antivascular endothelial growth factor drugs, and antioxidants can inhibit the occurrence of microvascular complications in diabetes.

**Conclusions:**

However, there has been no breakthrough in the treatment of diabetic microvascular complications. Therefore, prevention of diabetic microvascular complications is more important than treatment.

## 1 Introduction

Diabetes is a global health problem that affects people of all ages. With the change in production and lifestyle, the change in diet structure, and the intensification of population aging, the incidence rate, disability rate, and fatality rate of diabetes have increased year by year [1,2]. Diabetes, hypertension, and malignant tumors have been listed as the main diseases threatening human health and survival. The main clinical manifestations of diabetes patients are hyperglycemia, accompanied by polydipsia, polyphagia, polyuria, and body wasting [3,4]. Patients with diabetes are divided into type I diabetes and type II diabetes according to different states of insulin secretion insufficiency or insulin resistance [4].

Diabetes patients mainly show abnormal metabolism of sugars, lipids, and proteins, which belongs to the category of metabolic syndrome. The main harm of diabetes comes from chronic complications, microvascular complications include diabetic retinopathy (DR), diabetic nephropathy (DN), and diabetic peripheral neuropathy (DPN) [4,5,6]. DPN is a major cause of blindness, hemodialysis, and amputation, so the control of diabetic microvascular complications is the key to diabetes treatment [6]. This article reviews the epidemiology, molecular mechanism of occurrence and development, risk factors, and treatment methods of microvascular complications in diabetes, which provides a theoretical basis for more accurate elucidation of the pathogenesis and treatment of microvascular complications in diabetes.

## 2 Methods

A literature review was conducted in the PubMed database limited to English-language articles published using the terms: microvascular complication, epidemiology, molecular mechanism, risk factors, and treatment. Articles from 2019 through 2023 regarding pathogenesis and treatment of microvascular complications in diabetes were selected, reviewed, and summarized in this article with a focus on articles published within the previous 3 years. References from each article were also reviewed for additional relevant articles. The inclusion criteria are published peer-reviewed studies: quantitative (experimental and observational) or mixed method research design. The search results were imported into Endnote x7 and duplicates were removed. Two evaluators independently selected titles and abstracts using Rayyan. Publications identified by at least one reviewer as potentially relevant are searched in full-text form, and their qualifications are evaluated by two independent reviewers. When there are disagreements, a third reviewer can be arranged. Manually screen the list of references included in the publication to determine other publications that meet the inclusion criteria.

## 3 Results

### 3.1 Epidemiologic

#### 3.1.1 DR

DR is the most common diabetic microvascular complication and the first blinding eye disease in the working population [6,7]. The prevalence varies in different study populations: background stage DR is common in patients with diabetes who have had the disease for 20 years, proliferative diabetic retinopathy (PDR) occurs in about 70% of patients with type 1 diabetes mellitus (T1DM) after 30 years of disease [7,8]. The study found that among patients who developed diabetes before the age of 30 and were treated with insulin, the prevalence of DR Was 71%, the cumulative 4-year prevalence was 59%, and 11% of patients progressed to PDR [8,9]. Among patients who developed diabetes after age 30 and did not use insulin, the prevalence of DR Was 39%, the cumulative 4-year prevalence was 34%, and the progression to PDR was 3% [10].

#### 3.1.2 DN

DN is one of the most serious diabetic microvascular complications and the primary cause of end-stage renal disease (ESRD). The 2007 National Kidney Foundation clinical practice guidelines for chronic kidney disease, NKF-KDOQI proposes to use the name “diabetic kidney disease (DKD)” instead of “diabetic kidney disease” in clinical practice [11,12]. DKD usually presents as an increase in microalbuminuria in the urine, which then progresses to macroalbuminuria and renal failure. British prospective diabetes study (United Kingdom prospective diabetes study and UKPDS) is displayed in the 10 years of duration of diabetes patients in about 25% of the Ⅱ diabetes type 2 diabetes mellitus. Patients with T2DM develop microalbuminuria or more severe DKD [10,11,12,13]. It is estimated that approximately 50% of patients develop the microalbuminuria stage within 19 years of diagnosis of diabetes, and 2–3% of patients progress to the next severe stage each year [13,14].

#### 3.1.3 DPN

DPN is the most common and difficult complication to diagnose and manage. Its clinical manifestations are varied and can involve the body and autonomic nervous system [14]. Due to the existence of different evaluation methods and criteria, the prevalence of DPN varies greatly among different studies. In the European T1DM Complications study, 28% of T1DM patients had DPN [14,15]. A similar incidence was reported in T1DM or T2DM, with a prevalence of 32.3%. Pittsburgh Epidemunidogy of diabetes complications study, Pittsburgh Epidemunidogy of Diabetes Complications Study, PEDC, and the diabetes control and Complications Trial (DCCT) used the same criteria and had a higher prevalence [15]. In the Seattle Prospective Study of Diabetic Foot Disease, 50% of participants were found to have peripheral sensory nerve damage at baseline. The prevalence of diabetic neuropathy in multi-center outpatient clinics in China was 17.2% [16].

The prevalence of diabetic microvascular complications varies among different study populations. After 30 years of age, the prevalence of DR is 20–40%, and the prevalence of PDR is 5–10%. The prevalence of DKD ranges from 20 to 40%, with 2–3% of patients progressing to the next stage of severity each year [15,16,17].

### 3.2 Molecular mechanism

#### 3.2.1 DR

DR has become an important factor affecting patients’ quality of life. At present, retinopathy has been systematically studied and the molecular mechanism of its pathogenesis has been analyzed [16,17]. In the study of the protective effect of Purendan ultramicro powder on retinopathy of STZ-induced glycosuria rats and its effect on the NF-κB signaling pathway, it was found that PRD may have a protective effect on the retina of diabetic rats by specifically blocking the AGEs/RAGE/NF-κB signaling pathway [17,18]. Previous studies have also confirmed that the Shuangdan Mingmu capsule can play a role in the treatment of DR By regulating the Ras-Raf-1-MEK-ERK pathway, and CTGF can also effectively delay the process of retinal fibrosis through exogenous regulation and the balance between the level of vascular growth factor [14,16,17]. The study found that lowering TLR4 levels may improve obesity and insulin resistance in patients with type 2 diabetes, and targeted inhibition of TLR4 may be a potential target for the treatment of type 2 diabetes and its chronic complications [17,18,19]. Based on the analysis of PD1/PDL1 signaling pathways in the Damien treatment proliferation stage of DR study confirmed that the mechanism of Damien drinks Chinese traditional medicine preparation can improve the proliferation period diabetes complications in patients with retinal visual acuity level, and the process may be through inhibiting patient PD1/PDL1 signal transduction protein expression and activation to restore the function of mononuclear cells, The monocytes are transformed to inhibit inflammation and inhibit the expression of related inflammatory factors [18,19]. In the study of the protective effect and mechanism of silencing information regulatory factor associated enzyme 1 (SIRT1) regulating p38MARK signaling pathway on retinal ganglion cells in DR rats, it was also found that in DR models, SIRT1 expression was up-regulated and inhibited the apoptosis of RGCs [19]. The anti-apoptotic mechanism of RGCs in DR may be related to its inhibition of p38MARK expression.

Although the molecular mechanism of DR has been extensively studied, the root cause of DR has not been fully elucidated. DR is the main cause of clinical blindness in diabetic patients, and early intervention is particularly important for the development of retinopathy and the reduction of the blinding rate [18,19,20].

#### 3.2.2 DN

DN has become the most common cause of ESRD. With the increasing incidence of diabetes, the number of DN patients has shown an obvious upward trend [15,16,17,19]. According to the different responses to insulin, diabetes can be divided into type I diabetes and type II diabetes: the proportion of type I diabetes complicated with DN is about 35%, while the proportion of type II diabetes complicated with DN is about 22% [18,19]. In the study of regulating the renal inflammation in rats with DN through the TLR4/NF-κB signaling pathway, it was found that polydatin has an obvious protective effect on the renal inflammation caused by DN. The mechanism of action may be related to regulating the TLR4/NF-κB signaling pathway and reducing the content of inflammatory factors in kidney tissue [16,17,18,20]. And play anti-inflammatory and protective effects. In the study analyzing the effects of combined treatment of sagliptin on inflammatory factors and mononuclear cell PI3K-Akt pathway in elderly DN patients, it was also confirmed that the treatment of sagliptin can significantly reduce Hb A1c, 24 h UPro, and Cr in elderly DN patients, thus improving renal function [18,19]. At the same time, it can reduce TNF-α, IL-6, and IL-1 in peripheral blood, relieve peripheral inflammatory immune response, and significantly upregulate the phosphorylation levels of PI3K and Akt in peripheral blood mononuclear cells. When improving peripheral blood hyperglycemia, xagliptin can further restore the phosphorylation and activation levels of PI3K/Akt and participate in inhibiting inflammatory reactions [6,7,8,18]. Some scholars have also confirmed that the activation of the TGF/(Smad) signaling pathway and p38MARK signaling pathway in DN patients may be one of the main causes of kidney inflammation, and the elevated level of urine (1-MG protein) is a direct manifestation of impaired glomerular function, and the above indicators can be used as monitoring indicators for severe microangiopathy in diabetic patients [17,18,19]. In the study of regulating the renal inflammation in diabetic DN rats through the TLR4/NF-κB signaling pathway, it was also found that polydatin has a significant protective effect on the renal inflammation caused by DN, and its related mechanism may be related to regulating the TLR4/NF-κB signaling pathway and reducing the content of inflammatory factors in renal tissue [19,20]. And play an anti-inflammatory and protective role. Previous studies have also found that AQP5 may participate in the pathogenesis, development, and deterioration of DN, and the AC–cAMP–PKA pathway has a significant negative regulatory effect on the regulation of aquaporin 5 in renal tissue [20,21]. Previous studies have also confirmed that rhein can improve insulin resistance and inhibit mesangial cell apoptosis by regulating the expression of p-JNK and PPAR, thus protecting the kidney. Moreover, the classical signal transduction pathway mediated by IL-6 through IL-6 binding receptor protein and the transitional transduction pathway mediated by IL-6 through soluble IL-6 receptor have been shown to participate in the pathogenesis of DN [19,20,21].

The pathogenesis of DN may be related to a variety of intracellular signaling pathways, and a signaling pathway may be involved in the regulation of one or several action targets. Therefore, multi-signaling pathways should be analyzed *in vivo* and *in vitro* to provide a reference for further elucidating the pathogenesis of DN [21].

#### 3.2.3 DPN–diabetic foot

Diabetic foot is a kind of lower extremity infection ulceration and deep tissue destruction caused by diabetic patients complicated with nerve disease and various degrees of peripheral vascular lesions. Diabetic foot is a systemic disease with surgical symptoms such as extremity ulceration and bacterial infection, as well as clinical manifestations of medical diseases [21]. Diabetic foot is a serious stage in the development of diabetes, which seriously threatens the health of patients, and is also one of the main causes of disability and death of patients [21,22].

The treatment of diabetic feet is usually treated with ulcer therapy, infection therapy, and Charcot joint therapy, but the results are usually not satisfactory [22]. Therefore, the analysis of the molecular mechanism of the onset of diabetic foot has important practical significance for early prevention and treatment. Previous studies have also confirmed that the downregulation of Wnt/β-catenin pathway may lead to pathologic refractory ulcers in diabetic patients, and Simiayongan Decoction can also promote the healing effect of diabetic ulcers (such as diabetic foot) by regulating the level of Wnt/β-catenin signal transduction pathway related protein molecules [21,22,23]. In addition, MEBT/MEBO may dynamically regulate the expression levels of vascular conversion factor β1 and P-smad3 protein molecules, to promote the healing effect of diabetic foot ulcers in diabetic patients. In the study of the effect of peripheral blood mesenchymal stem cell transplantation on angiogenesis phosphatidylinoinositide 3-kinase (key molecule of PI3K signaling pathway)/protein kinase B signaling pathway in diabetic foot rats, it was found that peripheral blood mesenchymal stem cell transplantation can promote the proliferation and differentiation of endothelial cells by regulating the PI3K-AKT signaling pathway [22,23,24]. It can promote the formation of neovascularization in diabetic foot rats. In the analysis of the effects of peripheral blood mesenchymal stem cell transplantation on the angiogenic HIF-1/vascular endothelial growth factor (VEGF) pathway and miR-210 expression in diabetic foot rats, it was also found that peripheral blood mesenchymal stem cell transplantation may promote the proliferation and differentiation of endothelial cells and promote the formation of neovascularization by regulating the HIF-1/VEGF pathway and miR-210 expression [23,24,25]. The analysis of changes in the expression of the Wnt/β-catenin signaling pathway in diabetic ulcers also confirmed that the downregulation of the Wnt/β-catenin pathway may lead to the refractory diabetic ulcers, and the downregulation of this pathway may be derived from the decreased expression of Rspo-3 protein [17,18,24,25].

Diabetic foot is the most common microvascular disease of diabetes, but the current treatment measures are not satisfactory. Therefore, it is necessary to constantly explore the pathogenesis of diabetic foot and actively control the related pathogenesis factors and molecular mechanisms, so as to give priority to prevention and fundamentally reduce the risk of diabetic foot [26].

### 3.3 Risk factors and interventions

#### 3.3.1 Hyperglycemia

Hyperglycemia is the most important risk factor for the development of diabetic microvascular complications. The DCCT study found that in T1DM patients aged 13–39 years with a disease course of 1–15 years, intensive insulin therapy can effectively delay the appearance and progression of DR [26,27]. For patients without DR at baseline, the intensive treatment group reduced the risk of developing DR by 76% compared with the usual treatment group. For those with mild DR at baseline, the intensive treatment group had a 47% lower risk of DR progressing to PDR or severe NPDR compared to the usual treatment group [25,26,27]. The DCCT study also showed that intensive blood glucose control reduced the prevalence of microalbuminuria by 29% after the first year of treatment. In the secondary prevention cohort, intensive treatment reduced the risk of microalbuminuria progression by 43% and clinical albuminuria progression by 56% [26,27,28]. This effect of enhanced glycemic control was still seen at 4 and 7–8 years of follow-up. The study found that further control of HBA1c to 6.5% still reduced the risk of worsening kidney disease by 21%. Similarly, intensive treatment at 5-year follow-up reduced the development of clinical neuropathy by 64%, abnormal nerve conduction, and abnormal autonomic nervous system function changes by 44 and 53% compared with conventional treatment [27,28]. The SHDCS study in the Chinese population found that patients with HBA1c >7.5% had a 1.52-fold increased risk of microalbuminuria compared with patients with HBA1c <7.5%. However, the study did not find an effect of HBA1c on renal insufficiency in diabetic patients [20,28,29].

#### 3.3.2 Duration of diabetes

The course of diabetes is the main risk factor for microvascular complications of diabetes. The PEDC study evaluated 657 T1DM patients with childhood onset (0–17 years) and found that nearly all patients with diabetes >14 years, of course, developed background stage DR [17,18,19,29]. Among those with diabetes more than 25 years old, three-quarters of those with DR aged 18–29 years and more than half of those over 30 years old had PDR. The prevalence of PDR in the Wisconsin Epidemiological Study of Diabetic Retinopathy increased gradually with the duration of the disease (<5 years for diabetes, no PDR, 15% for 15 years for diabetes, and 67% for 35 years for diabetes) [27,28,29]. The SHDCS study showed that for every 5-year increase in the duration of diabetes, the risk of developing DR Increased by 1.2 times. Similarly, the UKPDS study found that the occurrence and progression of DKD were also related to the course of the disease: the proportion of annual progression from microalbuminuria was 2%, the proportion of progression from microalbuminuria to macroalbuminuria was 2.8%, and the proportion of progression from macroalbuminuria to elevated serum creatinine and renal replacement therapy was 2.3%; The proportion of patients with microalbuminuria for 10 years was 24.9% [14,15,16,17,29,30]. The proportion of patients with macroproteinuria was 5.3 and 0.8% on elevated serum creatinine and renal replacement therapy. There was a similar correlation between DPN and diabetes course. It was found that the frequency of subtypes of different types of neuropathy increased with the increase of time during 5–10 years [16,17,18,19,31]. Some scholars have also reported an increase in diabetic neuropathy with the increase in the course of the disease. The prevalence of diabetic neuropathy was 8.3% at baseline and increased to 41.9% after 10 years [31,32].

#### 3.3.3 Age of onset of diabetes

The age of onset of diabetes is another important risk factor for diabetic microvascular complications. Studies have shown a significant increase in diabetic microvascular complications, including DR, in puberty-onset diabetes patients with a 20-year course of disease compared with prepuberty-onset diabetes [30,31,32]. This change may be related to changes in hormone levels during puberty. The prepubertal course of diabetes was found to contribute little to DR. Although the prevalence of diabetes is higher in relatively older people, the prevalence of DR, especially in PDR, is low in people over 70 years of age when diabetes develops [17,18,29]. Similar to DR, the prevalence of overt DKD is significantly higher in adolescence-diagnosed diabetes than in preadolescence-diagnosed diabetes, and the risk of DKD is associated with the course of the disease after adolescence. Some scholars have found that the prevalence of microalbuminuria in T1DM increases significantly in the stage after adolescence [4,5,30].

Among the above risk factors, the course of diabetes and age of onset belong to uncontrollable risk factors. However, hyperglycemia, hypertension, smoking, and dyslipidemia are controllable risk factors, which can be controlled by intervention measures [28,29,30,32]. However, some patients continue to progress even after controlling risk factors. Therefore, to reveal the complexity of the pathogenesis of diabetic microvascular complications, genetic factors may also play an important role. In addition, the discovery of effective treatment is also an inevitable choice to inhibit its progress [29,30,31,32].

### 3.4 Treatment

Hyperglycotoxicity results in enhanced polyol pathway activity increased non-enzymatic glycosylation endproducts (AGEs) and protein kinase c. Increased activity of PKC and increased amino-hexose pathway were associated with diabetic complications [12,13,14,33]. Among them, high sugar induced the increase of mitochondrial reactive oxygen species in target cells, which was the initiating factor of the above four pathways. In addition to high glucose toxicity, diabetic hemodynamic abnormalities promote the occurrence and development of diabetic microvascular complications. Activation of the local renin–angiotensin system (RAS) plays an important role in the pathogenesis of diabetic microvascular complications [33,34].

Based on the above research results on the pathogenesis of diabetic microvascular complications, intervention is carried out targeting several molecular targets. It was found that RAS blockers, fibrates, statins, anti-VEGF drugs, and antioxidants can inhibit the development of diabetic microvascular complications [8,9,10,11,34].

#### 3.4.1 RAS blockers

RAS blockers include angiotensin-converting enzyme inhibitors and angiotensin receptor blockers (ARBs). The two drugs have been widely used in the clinical treatment of DKD, which can alleviate proteinuria, protect renal function, and inhibit the progression of DKD [34,35]. Concerning DR treatment, lisinopril has been found to inhibit the progression of DR in T1DM patients with normal blood pressure, with a rate of inhibition of primary progression up to 50% [16,17,35]. Recently, the study results of the DR candesartan trial have aroused concern that ARB Candesartan can reduce the incidence of retinopathy ≥grade 2 in T1DM patients by 18%. The incidence of retinopathy ≥grade 3 was reduced by 35% [33,34,35]. For T2DM patients, Candesartan resulted in a relative 13% reduction in the progression of retinopathy (primary outcome), although this result was not statistically significant; it resulted in a significant 34% increase in patients with improved retinopathy (secondary outcome) [35,36].

Alicylen is a representative renin inhibitor, and its application in diabetes has been in the clinical application research stage, mainly focusing on the protective effect on DN, diabetes complicated with hypertension, and target organs. It provides a new way for the adjuvant treatment of diabetes, but the long-term efficacy of the application in diabetic patients still needs to be explored in large-scale clinical trials [34,35,36].

#### 3.4.2 Fibrates

Peroxisouce proliferator-activated receptor-α (PPAR-α) is a member of the receptor superfamily and is involved in lipid metabolism and glucose homeostasis after activation by membrane ligands [36]. As an agonist of PPAR-α, fenofibrate has been widely recognized for its lipid-regulating effects and inhibition of the development of atherosclerosis, as well as its safety and tolerability. Renal PPAR-α was highly expressed in mesangial cells, proximal renal tubules, and collecting ducts [36,37]. In addition to the above-mentioned regulation of glucose and lipid metabolism, PPAR-α plays a role in regulating cell differentiation, cell cycle, extracellular matrix, and inflammatory pathways. A fenofibrate intervention and event lowering in diabetes (FIELD) study found that FIELD can significantly reduce the progression of albuminuria [15,16,17,37]. A subgroup analysis of the FIELD study found that fenofibrate reduced the deterioration of T2DM albuminuria by 24% compared to 11% with placebo. The subgroup analysis of the FIELD study found a 31 and 30% reduction in the number of cases of Fenofibrate DR requiring first laser treatment, respectively [36,37]. A subgroup analysis of patients who persisted to the end of follow-up, grading retinal status based on fluorescein fundus angiography results and using grade 2 progression in DR as the study endpoint, found that the risk of non-Nobel ennoblemen needing first laser therapy in this population was reduced by 70% [15,37,38]. However, the application of fenofibrate did not reduce the occurrence of DR, the progression of hard exudation, and the deterioration of vision, but the progression of the original retinopathy was significantly slow [38].

#### 3.4.3 Statins

Simvastatin can correct the production of reactive oxygen species induced by Ang II, enhance the biological activity of nitric oxide, reduce the production of nitrosine, and slow down the occurrence of DKD [38,39]. The Diabetes Control Cardiovascular Risk Group reported that fenofibrate combined with simvastatin reduced the progression of DR In T2DM patients by 40% for 4 years, and this effect was independent of the effect of blood glucose [39,40].

#### 3.4.4 Anti-VEGF

VEGF has a variety of biological functions, can increase vascular permeability, promote vascular endothelial cell migration, division, and proliferation, and thus promote angiogenesis [14,40]. Studies have shown that VEGF is closely related to the occurrence and development of diabetic microvascular complications. Intravitreal injection of anti-VEGF monoclonal antibody bevacizumab can effectively treat diabetic macular edema and can be used as an adjunctive therapy before advanced PDR surgery, greatly improving the surgical outcome [40,41].

#### 3.4.5 Antioxidant

There are many such drugs, such as α-lipoic acid, triphenylphosphate-bound coenzyme Q (MitoQ), L-propionyl-carnitine, LY333531, PJ34 and FP15 [36,37,38,40,41]. Currently, alpha-lipoic acid is routinely used in the clinic. Alpha-lipoic acid and its reduced form dihydrolipoic acid are powerful antioxidants that work by scavenging oxygen free radicals, interacting with other antioxidants, and inhibiting lipid peroxidation [16,17,18,19,41]. α-Lipoic acid can inhibit the activation of nuclear factor κB induced by hydrogen peroxide, tumor necrosis factor α, and advanced glycosylated end products to protect the function of vascular endothelial cells, increase the blood flow of neurotrophic vessels, promote nerve myelin formation and axon regeneration, repair damaged nerves, improve nerve conduction speed, and improve neuropathy symptoms [15,16,17,39]. Animal experiments show that α-lipoic acid has a good antioxidant effect, which can reduce the damage of free radicals and improve nerve function [14,15,16,17]. The statement of American Diabetes Association guidelines on diabetic neuropathy considers alpha-lipoic acid to be effective in the treatment of diabetic neuropathy, and many large evidence-based medical studies have subsequently proved that alpha-lipoic acid has a better peripheral neuroprotective effect [39,40,41].

In recent years, some scholars have also discussed the use of α-lipoic acid in the treatment of DKD. Clinical studies have shown that diabetic patients who were given 600 mg alpha-lipoic acid daily with urinary microalbumin creatinine ratio (ACR) <200 mg/L showed no progress in ACR after 18 months [40,41,42]. ACR in the control group was significantly increased (*P* < 0.05). Animal studies have shown that the combination of RAS blockers irbesartan and alpha-lipoic acid can more effectively control the growth of glomerular volume, the thickening of the sac basement membrane, glomerular basement membrane, and tubular basement membrane, as well as the expansion of mesangial matrix and tubular expansion [42,43].

#### 3.4.6 Others

PKC inhibitors, insulin sensitizers thiazolidinediones such as rosiglitazone, and peroxisome proliferation-activating receptor-δ (PPARδ) agonist GW501516 are also being studied to prevent the development of diabetic microvascular complications [43,44]. With the deepening of the understanding of the pathogenesis of diabetic microvascular, new methods for the treatment of diabetic microvascular complications are emerging [45]. However, there has been no breakthrough in the treatment of diabetic microvascular complications. Therefore, prevention of diabetic microvascular complications is more important than treatment [46].

## 4 Discussion

The incidence and disability rate of diabetes are becoming more and more serious with the intensification of diet structure and aging population. The occurrence of microvascular complications in diabetic patients has become an important killer that threatens their health and survival. DN, DPN, and DR are the most common and serious diabetic microvascular complications in clinical patients. Although the epidemiology, molecular mechanisms of occurrence and development, risk factors, and treatment of diabetic microvascular complications have been analyzed in this review, it should be noted that the occurrence of diabetes and its microvascular complications is not the result of a single signaling pathway. In future studies, different pathological models, *in vivo* and *in vitro* should be studied from multiple levels and perspectives, to provide reference for more accurately elucidating the pathogenesis and treatment of diabetic microvascular complications.
